# Deficient expression of DNA repair enzymes in early progression to sporadic colon cancer

**DOI:** 10.1186/2041-9414-3-3

**Published:** 2012-04-11

**Authors:** Alexander Facista, Huy Nguyen, Cristy Lewis, Anil R Prasad, Lois Ramsey, Beryl Zaitlin, Valentine Nfonsam, Robert S Krouse, Harris Bernstein, Claire M Payne, Stephen Stern, Nicole Oatman, Bhaskar Banerjee, Carol Bernstein

**Affiliations:** 1 Department of Cellular and Molecular Medicine, College of Medicine, University of Arizona, Tucson, AZ 85724, USA; 2 Department of Pathology, University of Arizona, Tucson, AZ 85724, USA; 3 Pathology Department, Saint Marys Hospital, 1601 West Saint Marys Road, Tucson, AZ 85745, USA; 4 Matrix Solutions Inc., 200, 150-13 Ave. S.W., Calgary, Alberta T2R 0V2, USA; 5 Department of Surgery, University of Arizona, Tucson, AZ 85724, USA; 6 Arizona Cancer Center, Tucson, Arizona 85724, USA; 7 Southern Arizona Veterans Affairs Heath Care System, Mail Stop 0-151, 3601 S. 6th Ave., Tucson, Arizona 85723, USA; 8 Biomedical Diagnostics and Research, 625 S. Plumer Ave, Tucson, AZ 85719, USA; 9 Department of Medicine, University of Arizona, Tucson, AZ 85724, USA

**Keywords:** "Colon cancer", "DNA repair", Pms2, Ercc1, Xpf, Ku86, "Genomic instability", Cancerization, "Field defect", Mutation

## Abstract

**Background:**

Cancers often arise within an area of cells (e.g. an epithelial patch) that is predisposed to the development of cancer, i.e. a "field of cancerization" or "field defect." Sporadic colon cancer is characterized by an elevated mutation rate and genomic instability. If a field defect were deficient in DNA repair, DNA damages would tend to escape repair and give rise to carcinogenic mutations.

**Purpose:**

To determine whether reduced expression of DNA repair proteins Pms2, Ercc1 and Xpf (pairing partner of Ercc1) are early steps in progression to colon cancer.

**Results:**

Tissue biopsies were taken during colonoscopies of 77 patients at 4 different risk levels for colon cancer, including 19 patients who had never had colonic neoplasia (who served as controls). In addition, 158 tissue samples were taken from tissues near or within colon cancers removed by resection and 16 tissue samples were taken near tubulovillous adenomas (TVAs) removed by resection. 568 triplicate tissue sections (a total of 1,704 tissue sections) from these tissue samples were evaluated by immunohistochemistry for 4 DNA repair proteins. Substantially reduced protein expression of Pms2, Ercc1 and Xpf occurred in field defects of up to 10 cm longitudinally distant from colon cancers or TVAs and within colon cancers. Expression of another DNA repair protein, Ku86, was infrequently reduced in these areas. When Pms2, Ercc1 or Xpf were reduced in protein expression, then either one or both of the other two proteins most often had reduced protein expression as well. The mean inner colon circumferences, from 32 resections, of the ascending, transverse and descending/sigmoid areas were measured as 6.6 cm, 5.8 cm and 6.3 cm, respectively. When combined with other measurements in the literature, this indicates the approximate mean number of colonic crypts in humans is 10 million.

**Conclusions:**

The substantial deficiencies in protein expression of DNA repair proteins Pms2, Ercc1 and Xpf in about 1 million crypts near cancers and TVAs suggests that the tumors arose in field defects that were deficient in DNA repair and that deficiencies in Pms2, Ercc1 and Xpf are early steps, often occurring together, in progression to colon cancer.

## Background

### Field defects

The term "field cancerization" was first used in 1953 to describe an area or "field" of epithelium that has been preconditioned by (at that time) largely unknown processes so as to predispose it towards development of cancer [1]. Since then, the terms "field cancerization" and "field defect" have been used to describe pre-malignant tissue in which new cancers are more likely to arise, and the concept of field cancerization in clinical oncology has received increasing attention [2,3]. For example, colon cancer patients are at about 9% to 55% risk for development of a second colon cancer in the next 5 years after a first cancer is resected [4], while members of the general population have less than a 1% risk of developing a colonic adenocarcinoma in this period.

### Field defects in the colonic mucosa

Field defects in the colonic mucosa probably arise by natural selection of a mutant or epigenetically altered cell among the stem cells of a crypt. The stem cells of a human crypt consist of about 10-20 cells at the base of a crypt in an area designated a stem cell niche [5,6]. With natural selection, a mutant or epigenetically altered stem cell may replace the other stem cells in a crypt, in a process called niche succession [7]. Genetic instability or a mutator phenotype, due to loss of DNA repair or loss of apoptosis competence, would accelerate this process [8]. If, among the stem cells in a colonic crypt, a cell acquires an advantage through a mutation or an epimutation, it will tend to expand clonally at the expense of neighboring stem cells. This process may give rise to "crypt conversion," whereby cells with a mutant or epigenetically altered genotype replace the other cells in the stem cell "niche" and generate an altered genotype for the entire cell population of a colonic crypt [9,10]. A human colonic crypt is shaped like a test-tube and consists of about 2,500 to 5,000 cells [11], being about 85 to 106 cells in length and about 29-43 cells in circumference [12].

**Figure 1:**
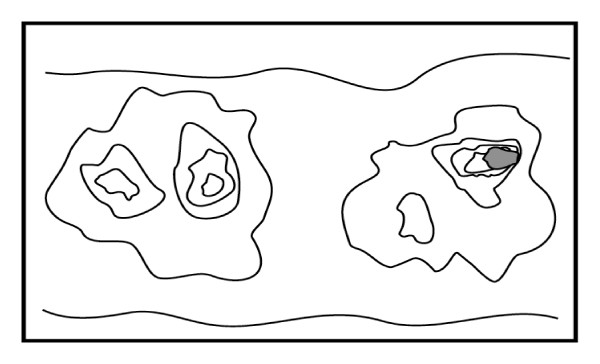
**Schematic diagram of colonic mucosa indicating progression of a field defect to colon cancer**. The gray area within the right-hand set of irregular concentric areas indicates a colon cancer. The outermost irregular concentric areas indicate initial defects with a selective advantage. A next smaller concentric area indicates a secondary mutation or epimutation giving a further selective advantage, while still smaller areas indicated further mutations or epimutations with still further selective advantages.

After one crypt is converted to a mutated or epigeneticaly altered crypt, a field defect may be formed by successive crypt fissions [10]. Thus, a patch of abnormal tissue may arise (a patch of many neighboring crypts in the epithelium of the colon, represented by the outermost irregular rings in Figure 1). Within a patch, a second such mutation or epigenetic alteration may occur so that a given crypt acquires an advantage compared to other crypts within the patch, and this crypt may expand clonally forming a secondary patch within the original patch. Within this new patch, the process may be repeated multiple times until a malignant stem cell arises which clonally expands into a cancer (dark area in Figure 1). If this is the general process by which sporadic colonic adenocarcinomas arise, then colonic adenocarcinomas generally should be associated with, and be preceded by, fields of increasing abnormality reflecting the succession of premalignant events. The most extensive regions of abnormality (the outermost irregular rings in Figure 1) would reflect the earliest events in carcinogenesis.

### Genomic instability in cancer

Colon cancer is a disease associated with genomic instability [13]. Colon cancers have between 49 to 111 non-silent mutations, with an average of 15 of those mutations being "drivers" of carcinogenesis, and the remaining ones being "passengers" [14]. However, there is no clear common pattern of mutations in different colon cancers. In addition, there is a median of 9 copy number changes (homozygous deletions and amplifications of genes) per colon cancer [15]. But it has been a puzzle as to how this instability originates. However, we note that if an early event in the development of a field defect were loss of DNA repair capability, this would allow DNA damages to escape repair and could give rise to the increased mutations and chromosome aberrations that are characteristic of cancer.

### Reactive oxygen species and their specific targets

As reviewed by Ziech et al. [16], reactive oxygen species (ROS) play an important role in progression to cancer. ROS cause reduction in expression of at least two key DNA repair enzymes, Ercc1 and Pms2, *in vitro*. Chang et al. [17] showed that elevated ROS degrade Pms2 but not two other mismatch repair proteins (Mlh1 and Msh2) suggesting that Pms2 is a specific target of oxidative stress.

Exposure of human cells to a non-toxic level of H_2_O_2 _caused a 5-fold decrease in expression of Ercc1, possibly by direct oxidative attack of the protein [18]. In contrast, expression of several other nucleotide excision repair proteins (e.g. Xpa, Xpc, Ercc4 and Ercc5) increased 2 to 4.5-fold, suggesting that Ercc1 is also a specific target of oxidative stress. Nucleotide excision repair capacity decreased to less than 50% by the H_2_O_2 _treatment in a manner that correlated with loss of Ercc1 [18].

### Roles of Pms2 and Ercc1

Due to the important role of ROS in cancer and the indication that Pms2 and Ercc1 are specific targets of ROS, we directed our attention to evaluating whether Pms2 and Ercc1 (and Xpf, the pairing partner of Ercc1) were systematically reduced in progression to colon cancer. In a previous preliminary study we found that tissue samples from patients with large tubulovillous adenomas or adenocarcinomas had reduced Pms2 expression in cell nuclei at the bases of crypts (including the stem cell regions) near these tumors. This reduction was associated with reduced apoptosis competence [19], as might be expected since Pms2 is also needed for apoptosis [20]. Ku86 was added to our study due to Rigas et al. [21] indicating that a reduction in Ku86 could be important during human colon carcinogenesis.

Germline mutations in DNA mismatch repair genes give rise to hereditary non-polyposis colon cancer, which accounts for about 2.2% of colon cancer [22]. In the human DNA mismatch repair system, Pms2 interacts with Mlh1 to form the MutLα heterodimer, and Pms2 is unstable in the absence of Mlh1 [23]. The Mlh1/Pms2 protein complex, together with other components of the mismatch repair system, corrects single base mismatches and small insertion/deletion loops that occur during DNA replication.

In mice, the Pms2/Mlh1 heterodimer is also required for the normal apoptotic response to DNA damage [24]. Pms2 is a dual role protein, needed for DNA repair and also for apoptosis (reviewed in [20]). Pms2 mediates the apoptotic response through interaction with p73, a p53-related protein, and p73, like p53, is an activator of apoptosis [25]. Decreased apoptosis capability in Pms2 deficient mice allows cells with DNA alkylation damages to survive, leading to increased mutation [24]. Mice with deficient Pms2 have a 100-fold elevated level of spontaneous mutation [26].

The human Ercc1/Xpf complex is a DNA endonuclease essential for one of the two incision steps of nucleotide excision repair (NER), a DNA repair pathway that removes helix-distorting DNA damages. Ercc1/Xpf endonuclease incises the DNA on the 5' side of the damaged site. The NER repair system can repair oxidative DNA damages such as 8-oxoguanine [27]. The Ercc1/Xpf complex also facilitates repair of DNA double-strand breaks [28] and inter-strand crosslinks [29], presumably through the process of homologous recombinational repair [30]. Mice deficient for Ercc1 show accelerated mutation accumulation [31]. Mice with skin-specific Ercc1 inactivation are hypersensitive to UV-induced skin cancer [32].

## Results

### Expression of Pms2, Ercc1 and Xpf in tissue samples from individuals who never had a colonic neoplasm

The levels of expression of DNA repair proteins Pms2, Ercc1 and Xpf were evaluated by immunohistochemistry (IHC) in sequential tissue sections from individuals who never had a colonic neoplasm, and thus are at low risk of colon cancer. The 4 micron tissue sections were placed on sequential slides and separately immunostained for Pms2, Ercc1 and Xpf. Crypts are usually about 60 to 80 microns in width, when measured through crypts for which the crypt lumen is visible, so that the cut through the crypt is through the central region of the crypt. Since the tissue sections were 4 microns thick, a series of about 15 sequential tissue sections could be cut through the same crypt and be displayed on separately immunostained slides.

**Figure 2:**
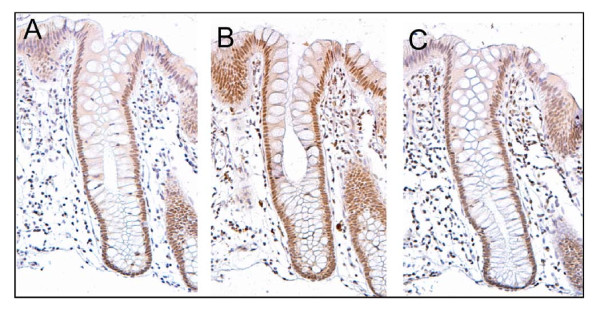
**Sequential sections of the same crypt with high expression of Pms2 (A), Ercc1 (B) and Xpf (C)**. This crypt, from the biopsy of a 58 year old male patient who never had colonic neoplasia, shows high expression (brown) in absorptive cell nuclei throughout most of the crypt for each of the proteins. Note that Pms2 and Xpf expression (in panels A and C) are each reduced or absent in the nuclei of cells at the top of the crypt and within the surface of the colonic lumen between crypts. Images taken at 200×.

Figure 2 shows the same crypt immunostained with antibodies to Pms2, Ercc1 and Xpf. The cells of this crypt had high expression of Pms2, Ercc1 and Xpf in the nuclei of most of the cells of the crypts.

While Pms2 is expressed at high level in nuclei of absorptive cells throughout the major portion of the crypt, at the open top of the crypt, near the colonic lumen, there is reduced nuclear expression of Pms2 (Figure 2, panel A). This pattern of expression of Pms2 is typical for crypts within the colonic epithelium of patients at low risk for colon cancer.

A similar pattern of nuclear expression is seen for Xpf in the epithelium of patients at low risk for colon cancer, except that Xpf is sometimes expressed at high levels, and sometimes at low levels, in areas of the colonic epithelium between crypts. The pattern for Ercc1 in low risk patients, however, is to have high nuclear expression in all absorptive cells, both within the crypts and along the epithelium of the colonic lumen.

### Expression of Pms2, Ercc1 and Xpf in histologically normal tissue samples taken from colonic resections that include an adenocarcinoma

**Figure 3:**
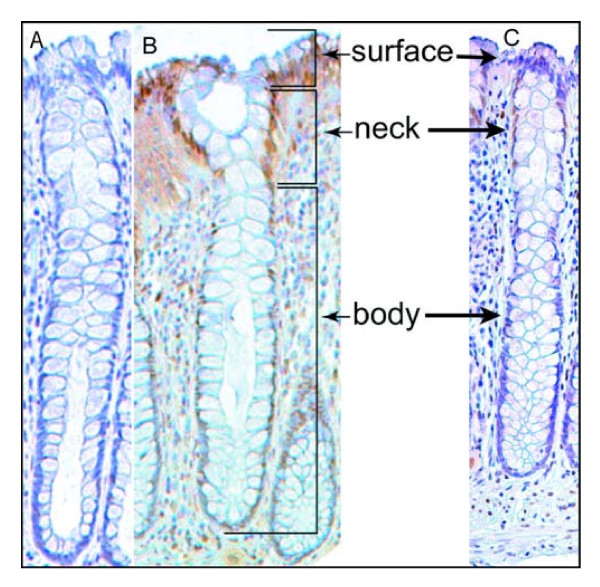
**Single crypts having deficient expression for Pms2 (A), Ercc1 (B) or Xpf (C)**. These crypts are from a histologically normal area of a colon resection of a male patient who had an adenocarcinoma in the sigmoid colon. When Pms2 is deficient, typically all the cells of the crypt have low or absent expression for Pms2 (A). When Ercc1 is deficient, cells of the "body" have reduced or absent expression of Ercc1 but cells of the "neck" and "surface" usually have high expression for Ercc1 (B). When Xpf is deficient, cells of the body have reduced or absent expression of Xpf, but cells of the neck region often have high expression of XPF. Cells at the surface may have reduced expression (C) or may have high expression for Xpf (not shown here but see Figure 4). Images taken at 200×.

For convenience in further discussion, we refer to three regions of the colonic crypt and adjacent mucosa as illustrated in Figure 3. These regions are the main body of the crypt, a region near the colonic lumen referred to as the "neck", and the surface epithelium of the colonic lumen that is present between crypts. The three crypts shown in Figure 3 were each located about 10 cm from a colonic adenocarcinoma. We evaluated nuclear staining of absorptive cells of a crypt as being at levels 0, 1, 2, 3, or 4, with 0 indicating no detectable staining, 1 indicating just barely detectable staining, 2 indicating low but clearly present staining, 3 indicating moderately strong staining, and 4 indicating a very high level of staining. Scoring was performed with observations both in a Motic digital BA300 photomicroscope at 400×, and as digital images at 400× on a high contrast ratio computer monitor.

Figure 3 shows images taken at 200× (to allow entire crypts to be seen in a single view), but even at this reduced magnification we can see that, in panel A, staining for Pms2 within the crypt is at level 0. In Figure 3 panel B, staining for Ercc1 was largely at levels 0, 1 or 2 in the body of the crypt, while in the neck and the surface regions nuclear staining was at levels 3 or 4.

In Figure 3 panel C, staining for Xpf was at level 0 or 1 in the body of the crypt, level 2 at the neck of the crypt and level 0 or 1 at the surface.

When evaluating staining of absorptive cells in the bodies of individual crypts, or in crypt bodies overall in a tissue section, we designate levels 3 and 4 as high expression and levels 0, 1 and 2 as reduced expression.

**Figure 4:**
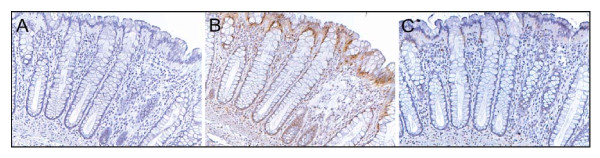
**Sequential tissue sections with all crypts having reduced "body" expression of Pms2 (A), Ercc1 (B) and Xpf (C)**. These crypts are from a histologically normal area of a colon resection of a male patient who had an adenocarcinoma in the sigmoid colon (same tissue as in Figure 3). There is high expression of Ercc1 at the neck and surface of the crypts (B). There is also some expression of Xpf at the neck of each crypt but not in the clonic lumen in this area of tissue (C). For Pms2 (A), there is reduced expression in the body, the neck and surface for all epithelial cells. Images taken at 200×.

A frequent finding was that tissue samples defective in one of the proteins Pms2, Ercc1 or Xpf were simultaneously defective in one or both of the other proteins. Figure 4 shows sequential sections of histologically normal tissue taken 10 cm away from an adenocarcinoma stained either for Pms2 (A), Ercc1 (B) or Xpf (C). In each case the crypts have reduced staining for the respective proteins in all cells in the bodies of the crypts. This contrasts with the strong pattern of staining observed in sequential sections from low risk individuals (Figure 2).

**Figure 5:**
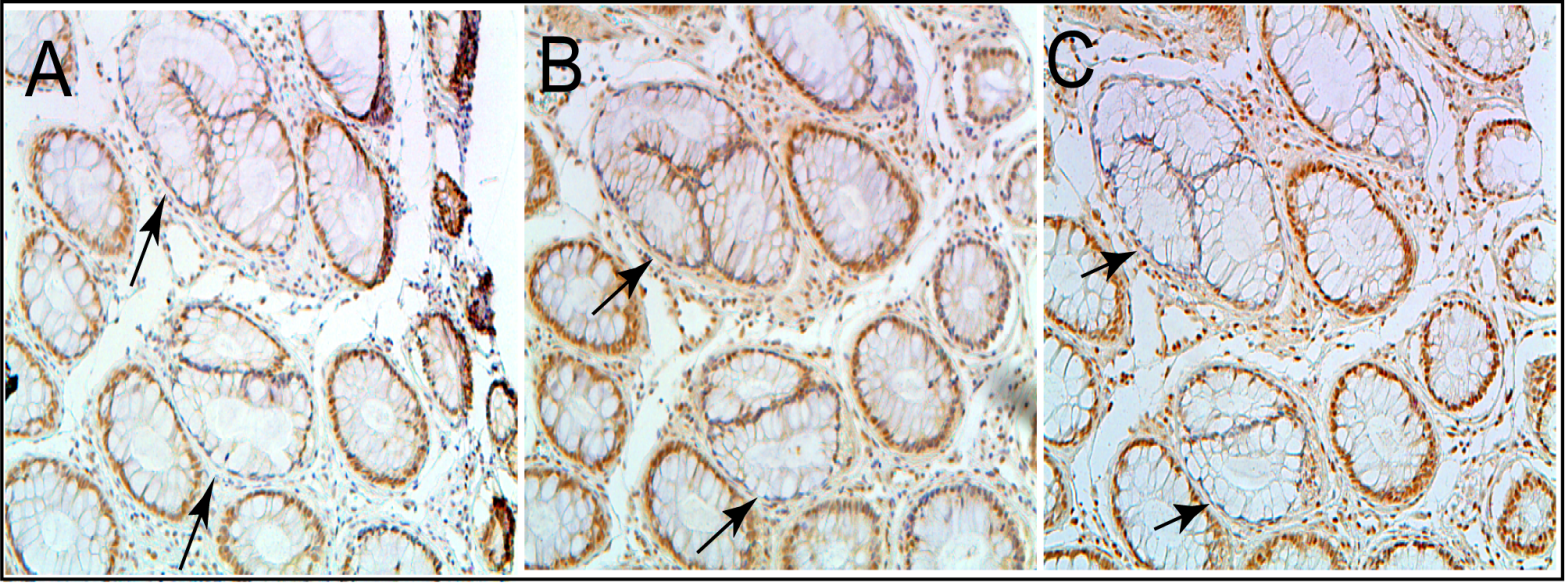
**Tissue with fissioning crypts stained for Pms2 (A), Ercc1 (B) and Xpf (C)**. These sequential sections were from histologically normal tissue marginal to a resected sigmoid adenocarcinoma of a female patient. There appear to be fissioning crypts in this area of tissue, indicated by arrows. These fissioning crypts have reduced expression for each of the three proteins, Pms2, Ercc1 and Xpf. Most of the other crypts in this area have high expression of Pms2, Ercc1 and Xpf in their cell nuclei. These fissioning crypts may constitute a small patch of DNA repair-defective crypts that are increasing in patch size by crypt fission. Images taken at 200×.

Figure 5 shows sequential cross sections through a group of colonic crypts stained for Pms2 (A), Ercc1 (B), or Xpf (C). The fissioning crypts (indicated by arrows) in these panels are each deficient in Pms2, Ercc1 and Xpf. These crypts illustrate a possible newly deficient stage in the formation of a field defect, expanding by crypt fission. The DNA repair protein deficiencies they show have likely relevance to progression to colon cancer.

**Figure 6:**
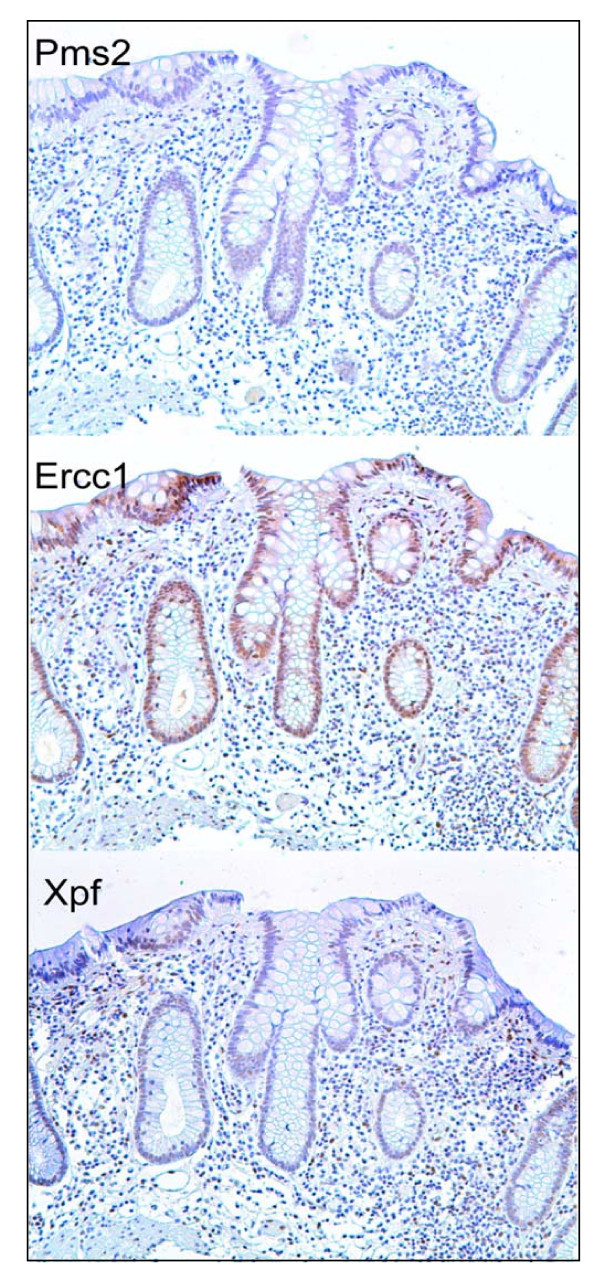
**Sequential tissue sections with discordant expression of Pms2, Ercc1 and Xpf**. These crypts are from the biopsy of a 71 year old female patient who had a 1 cm adenocarcinoma. While Pms2 and Xpf have reduced expression in this tissue area, Ercc1 has high expression. In this mucosal area, one crypt is apparently fissioning into 3 crypts. Images taken at 200×.

Figure 6 shows sequential sections through another group of colonic crypts stained for Pms2, Ercc1 or Xpf. The sections were from histologically normal tissue 10 cm from a colonic adenocarcinoma. The figure shows a crypt apparently fissioning into three crypts. There is high expression of Ercc1, but reduced expression of Pms2 and Xpf. This figure illustrates a common observation, that two of the three measured proteins may be simultaneously deficient, but not necessarily all three.

**Figure 7:**
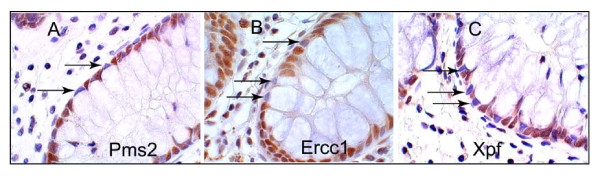
**Absorptive cells and goblet cells in crypts express DNA repair proteins differently**. These three panels show parts of single crypts from a biopsy of a male patient who had a 1.5 cm tubular adenoma, stained by IHC for Pms2 (A), Ercc1 (B) and Xpf (C). Goblet cells of the crypts have a large "balloon like" region containing mucin granules (cytoplasmic white areas under these staining conditions). The other cells in the crypts are absorptive cells. Arrows indicate some nuclei of goblet cells (on the outer edges of the crypts) that have reduced or absent expression for Pms2, Ercc1 and Xpf. Images taken at 1000×.

Absorptive cells and goblet cells are the two predominant types of cells in the colonic crypt epithelium. When evaluating cells of the body of a crypt for level of expression of Pms2, Ercc1 or Xpf, only absorptive cells were evaluated since their expression was consistently high in low risk patients. The goblet cells, each with a large "balloon like" region containing mucin granules (white areas under these staining conditions), often had low or absent expression for the protein of interest, even in relatively lower risk patients, as shown in Figure 7. The several thousand goblet cells of a crypt arose from the few stem cells at the base of the crypt, as did the absorptive cells of the crypt. The frequent lack of expression for Pms2, Ercc1 or Xpf in goblet cells appears to indicate a possible epigenetic alteration during their differentiation.

**Figure 8:**
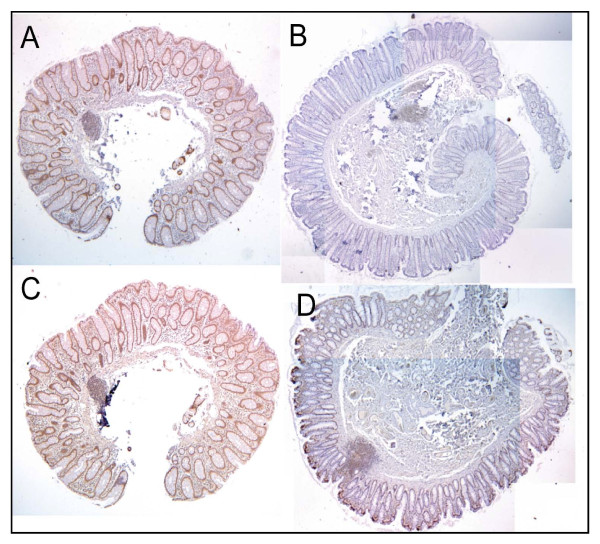
**Two pairs of entire tissue sections with consistent protein expression throughout all the crypts**. Panels A and C are sequential tissue sections from a biopsy taken from a 57 year old male patient with current tubulovillous adenomas and a history of colon cancer. Panels B and D are non-sequential tissue sections from a tissue sample taken from a resection from a male patient who had a carcinoma in the sigmoid. Panels A and B were stained for Pms2, panels C and D were stained for XPF. All crypts in each tissue section had similar levels of protein expression, with high (A, C) or low (B, D) protein expression. Images B and D were tiled, since the entire tissue section could not be captured in one field of view. Images taken at 40×.

When absorptive cells of a crypt near a colonic adenocarcinoma are deficient in expression of Pms2, Ercc1 or Xpf, they are also often similarly deficient in all of the crypts within the tissue section. A deficient crypt near a colon cancer is most often part of a large patch of deficient cells. To illustrate this, Figure 8A shows an entire tissue section, with about 40 crypts in the section, with normal high staining for Pms2 and Figure 8B shows an entire tissue section with about 100 crypts, all deficient for Pms2. Similarly Figure 8C shows an entire tissue section (sequential to that shown in panel A) with high staining for Xpf, and Figure 8D shows an entire tissue section, all with reduced staining for Xpf within the bodies of the crypts, although there is high staining along the surface of the colonic lumen.

### Expression of Ku86 in histologically normal tissue samples taken from colonic resections that include an adenocarcinoma

**Figure 9:**
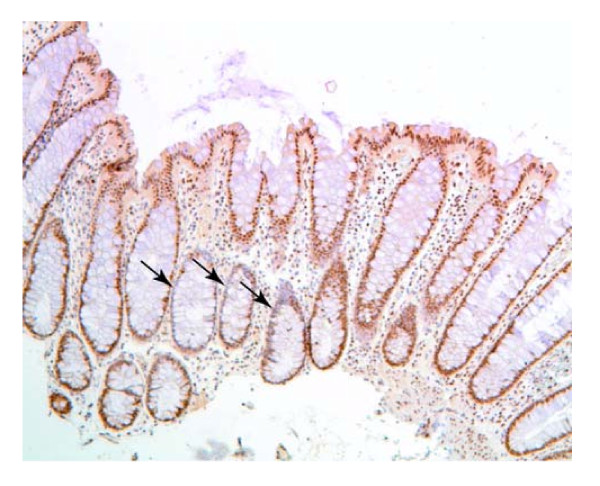
**A tissue section immunostained for Ku86**. A tissue section from a biopsy of a 53 year old male patient who never had a colonic neoplasia. A small patch of three crypts with reduced expression for Ku86 is shown by arrows.

As described above, the pattern of deficiency shown by Pms2, Ercc1 and Xpf is that, most often, when one or more of these proteins are reduced in expression, all the crypts of the tissue section are similarly deficient. That is, the patch size of the field defect is large. Ku86 deficient crypts, however, tend to occur in small patches of 1 to 3 crypts with reduced staining. Figure 9 shows a section of colonic mucosa that has a patch of 3 crypts with reduced expression of Ku86 and with all nearby crypts expressing high levels of Ku86.

### Semi-quantitative evaluation of Pms2, Ercc1, Xpf and Ku86 in histologically normal tissues and in colon cancers

To make a semi-quantitative estimate of the degree of deficiency of Pms2, Ercc1, Xpf and Ku86 in areas surrounding colon cancers, the level of expression of these proteins in histologically normal tissue of patients who never had a colonic neoplasia was first evaluated. Such individuals have about a 2.4% chance of developing an advanced colonic neoplasia within the next 5.5 years [33], and thus presumably have few genetic or epigenetic alterations leading to progression to colon cancer.

The absorptive cells in the "bodies" of crypts of an entire tissue section were evaluated for percent of cells with high level of expression of each of four DNA repair proteins Pms2, Ercc1, Xpf and Ku86. Pms2, Ercc1 and Xpf were evaluated in triplicate sequential 4 micron tissues sections of the same tissue samples. Ku86 tissue samples were from similar, but not identical, tissues to those evaluated for Pms2, Ercc1 and Xpf.

Tissue sections were prepared from 19 patients who never had any colonic neoplasia and were immunostained with antibodies to Pms2, Ercc1 and Xpf. Tissue sections from 10 such patients were immunostained with antibodies to Ku86. These minimal risk individuals were labeled category "A" and box and whisker plots were made of the percent of absorptive cells with high expression of the four proteins Pms2, Ercc1, Xpf or Ku86 in the colonic crypts.

Although the standard deviation is ordinarily used to decipher the spread of data when values follow a symmetrical distribution, box and whisker plots are preferred when data are asymmetrically distributed and contain outliers. In this type of plot, the median value is marked by a horizontal line within the box. The ends of the box mark the upper and lower quartiles, so the box, itself, spans the range of the two internal quartiles. The whiskers are the two lines outside the box that extend to the highest and lowest observations (except for the outliers). Outliers are indicated as individual points.

**Figure 10:**
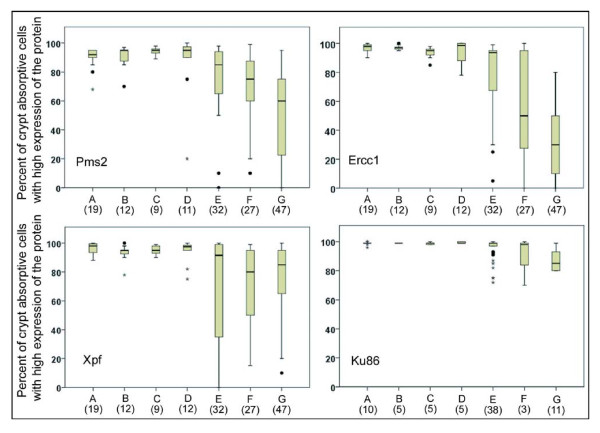
**Tissue samples evaluated with respect to expression of Pms2, Ercc1, Xpf or Ku86**. Entire tissue sections were evaluated with respect to all crypt absorptive cells seen in the tissue or within all cells of epithelial origin within a cancer. Pms2, Ercc1 and Xpf were evaluated in triplicate sequential 4 micron tissues sections of the same tissue samples. Ku86 tissue samples were from similar, but not identical, tissues to those evaluated for Pms2, Ercc1 and Xpf. Tissue samples from patients at different risks for colon cancer, or from colon segments resected because of colon cancer, were labeled A, B, C, D, E, F, G and the number of tissue samples, from different patients, evaluated for protein expression are shown in parentheses under their label. The tissue samples were from colonic biopsies from patients who had (**A**) never had an adenoma; (**B**) 1 to 3 adenomas < 1 cm; (**C**) advanced neoplasia with either adenoma > 1 cm, villous adenoma, or adenoma with dysplasia; (**D**) previous colon cancer; or from colonic resections with (**E**) tissue sample 1 to 10 cm distant from an adenocarcinoma; (**F**) area marginal to the tumor; or (**G**) epithelial origin cells within the adenocarcinoma.

As can be seen in Figure 10, for patients labeled A, who are at minimal risk for progression towards colon cancer, the median percents of absorptive cells with high expressions of Pms2, Ercc1, Xpf and Ku86 in their tissue sections were each above 90%, and at least 75% of all tissue sections of these lowest risk patients had more than 90% of the absorptive cells with high expression of all 4 proteins (values within the box plus within whiskers with higher values).

We were also interested in whether deficiencies in protein expression of Pms2, Ercc1, Xpf or Ku86 could be biomarkers of colon cancer risk within biopsies taken during colonoscopies. We stratified patients whose biopsies were obtained and who did have some level of colonic neoplasia into categories B, C, and D. Patients who had a current or previous record for a total of 1 to 3 adenomas that were less than 1 cm in diameter were labeled B. Patients with a current or previous record of an advanced neoplasia (with either adenoma > 1 cm, villous adenoma, or adenoma with dysplasia) were labeled C. Patients with a current or previous record of colon cancer were labeled D. These groups were comparable to the groups stratified by Lieberman et al. [33]. In the 5.5-year follow-up of more than 1,000 patients, Lieberman et al. [33] found that patients similar to our group B had a 6.1% chance, those similar to our group C had about a 16% chance, and patients similar to our group D had a 34.8% chance of developing an advanced colonic neoplasia within the next 5.5 years.

Figure 10 indicates that the median percent of absorptive cells with high expression of Pms2, Ercc1, Xpf and Ku86 in their tissue sections were each above 90% for the patients in groups B, C and D. Thus, these proteins would not make good biomarkers for risk of colon cancer.

There were also 32 tissue samples obtained at 1-10 cm from colon cancers (removed during resection) that were cut as triplicates and immunostained for Pms2, Ercc1 and Xpf, as well as 38 additional tissue samples obtained at 1-10 cm from colon cancers that were immunostained for Ku86. These were labeled E in Figure 10. In addition, 27 tissue samples obtained as samples of colon cancers had marginal tissue attached with histologically normal colonic morphology that were observed after the "colon cancer samples" were cut in triplicate and immunostained for Pms2, Ercc1 and Xpf, and 3 such tissue samples were cut and immunostained for Ku86. These marginal tissue samples were labeled F in Figure 10. A total of 47 samples of colon cancers (the 27 with marginal tissues included and 20 without marginal tissues) were cut in triplicate and immunostained for Pms2, Ercc1 and Xpf, plus a total of 11 colon cancers (3 with marginal tissues and 8 without marginal tissues) were sectioned and immunostained for Ku86. Tissues from within cancers were labeled G in Figure 10.

For the three tissue groups labeled E, F and G, the levels of expression of Pms2, Ercc1 and Xpf are much more broadly distributed and the median values are lower than in groups A-D. In contrast, for Ku86, the tissue groups labeled E and F do not show a lower median value than shown in groups A-D, and only group G, within the cancers shows a lower median value. For Ku86, the range in values is not as great as the range in values for Pms2, Ercc1 and Xpf in groups E, F and G. That is, there is less variability and much less loss of expression for Ku86 than for Pms2, Ercc1 and Xpf in comparable tissues.

In general, these results show that expression of Ercc1, Xpf and Pms2 is reduced and more variable (among tissue samples) in the colonic mucosa nearby or within tumors, compared to the colonic mucosa from biopsies taken during colonoscopies. Ku86 is less frequently reduced in expression in tissues near tumors, and has a more modest reduction within tumors than the other three DNA repair proteins evaluated.

### Expression of Pms2, Ercc1 and Xpf in pairwise associations

In Figures 4, 5 and 6 we illustrated the frequent finding that tissues are often simultaneously deficient for two or all three of the proteins Pms2, Ercc1 or Xpf. We next measured the frequencies with which pairwise combinations of these proteins (Pms2 & Ercc1; Pms2 & Xpf; Ercc1 & Xpf) are deficient in expression of both proteins together in the same tissue.

As noted above, the expression values for Pms2, Ercc1 and Xpf are asymmetrically distributed in tissues near or within tumors. On the other hand, the expression values of Pms2, Ercc1 and Xpf for the lowest risk group, group A, have an approximately symmetric distribution, and so the mean and standard deviation σ can be calculated. The mean expression level ± 2σ can be taken as the range in which 95% of the expression values are expected to fall. If the lowest risk group is taken as the normal group, then values for any other group that fall outside the range of the mean ± 2σ can be considered abnormal expression values.

**Figure 11:**
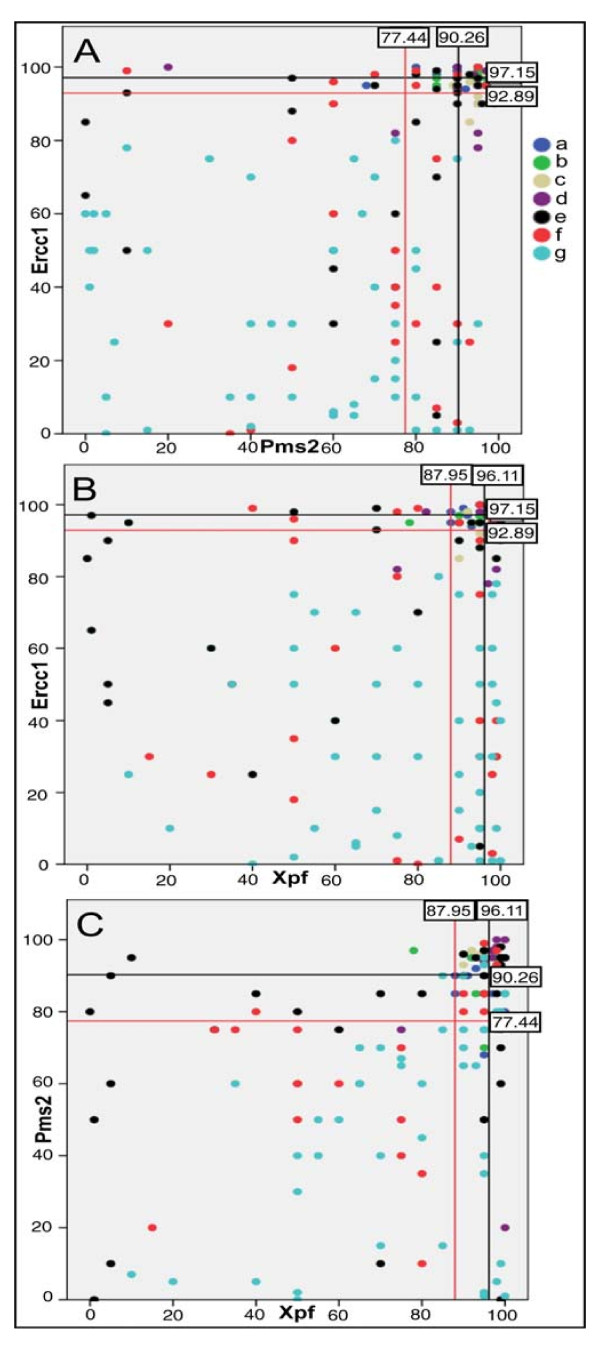
**Each dot represents the joint evaluation of a tissue sample for 2 DNA repair proteins**. The colors of the dots a, b, c, d, e, f, g represent tissues of groups A, B, C, D, E, F, G respectively, defined in Figure 10. Black lines represent mean values of expression of tissue sections from group A while red lines represent values of expression which are -2σ less than the mean values. Numbers in the small boxes show the mean and -2σ expression values. Dots that are below and to the left of the red lines have significantly reduced expression for both proteins being evaluated.

Figure 11(A) shows the expression values of Pms2 plotted against the expression values of Ercc1 for each sample. In Figure 11(A), the mean and -2σ values of Pms2 for the lowest risk group A patients are shown by two vertical lines towards the right of the diagram. The mean value (black line) was 90.26 percent high expression for all absorptive cells in the bodies of the crypts in a tissue section (the value shown in the small box at the top of the black line). Two standard deviations below this mean value (shown by the red line) was 77.44 percent high expression (shown in the small box at the top of the red line). All expression values to the left of the -2σ line can be regarded as representing deficient Pms2 expression. Likewise, the mean and -2σ values for Ercc1 expression are shown by two horizontal lines near the top of the diagram, with the mean value of 97.15 shown in the black line and the -2σ value of 92.89 shown in the red line. All values below the -2σ line can be regarded as representing deficient Ercc1 expression.

**Figure 12:**
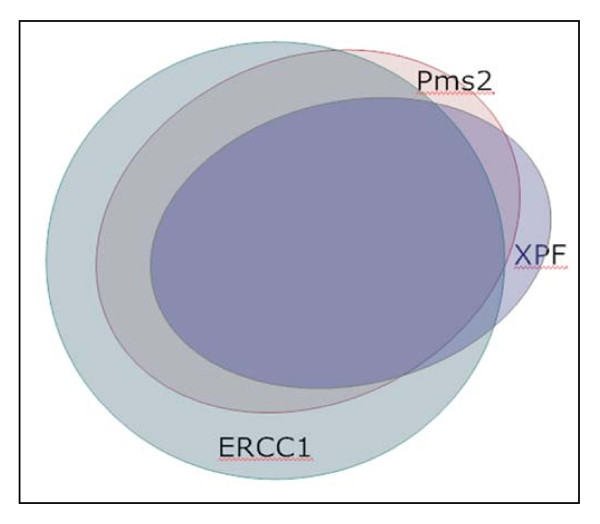
**A Venn diagram illustrating the level of concordance of deficiencies of Pms2, Ercc1 and XPF**. Entire tissue sections were evaluated with respect to all crypt absorptive cells seen in the tissue or within all cells of epithelial origin within a cancer. Pms2, Ercc1 and Xpf were evaluated in triplicate sequential 4 micron tissues sections of the same tissue samples.

There were 63 samples deficient for Pms2. Of these 63 samples, 56 (89%) of samples were also deficient for Ercc1. Overall, 85 samples jointly stained for Ercc1 and Pms2 were deficient for Ercc1. Of these, 56 (66%) were also deficient in Pms2 expression. Thus a higher percentage of Pms deficient samples were also deficient for Ercc1 (89%), than Ercc1 samples were deficient in Pms2 (66%). This could parallel the situation found by Nara et al. [34] where defects in Pms2, an apoptosis (and mismatch repair) gene, were selected for once Ercc1 was defective, but other apoptosis genes were alternatively selected for once Ercc1 was defective, as described further in the Discussion, below. The overlap between Pms2 and Ercc1 deficiencies is shown in the Venn diagram of Figure 12.

Figure 11(B) shows the expression values of Ercc1 plotted against the expression values of Xpf when sequential tissue sections were immunostained for Ercc1 and Xpf respectively. Overall, 81 tissue samples jointly stained for Ercc1 and Xpf were deficient in Ercc1. Of these, 43 (53%) were also deficient in Xpf expression. Also 53 samples were deficient for Xpf. Of these samples, 43 (81%) were also deficient for Ercc1. Thus a higher percentage of Xpf deficient samples were also deficient in Ercc1 (81%), than Ercc1 samples were deficient in Xpf (53%). These overlapping deficiencies are illustrated in Figure 12.

Figure 11(C) shows the expression values of Pms2 plotted against the expression values of Xpf for each sample when both were available for the samples. Overall, 62 samples are deficient for Pms2. Of these, 41 samples (66%) were also deficient for Xpf. There were 52 samples deficient for Xpf. Of these, 41 (79%) were also deficient in Pms2. The overlap between Pms2 and Xpf is shown in Figure 12.

### Extent of the field defects with reduced expression of Pms2 and Ercc1

**Figure 13:**
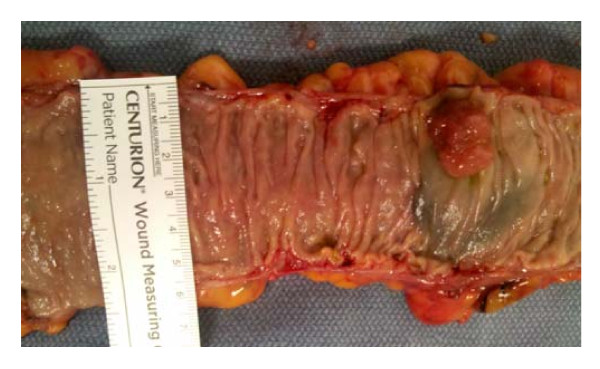
**Part of a sigmoid area colon resection, opened longitudinally, and showing a colon cancer**. Tissue samples were obtained from such freshly resected colon segments. A ruler was placed across the opened colon segment to determine the inner circumference of the opened colon segment.

We next consider the extent of the field defects that are deficient in Pms2 and Ercc1 in colon segments that were resected, due either to the presence of colon cancer or due to large tubulovillous adenomas. Crypts were scored in tissue sections for percent of crypts with high expression of Pms2, Ercc1 or Ku86. Data derived from 8 patients with a colonic adenocarcinoma and 8 patients with a large tubulovillous adenoma (TVA) are shown in Table 1. Where possible, we took 6 tissue samples from both sides of a colon cancer at distances of 1, 3 and 10 cm to the proximal side and 1, 3 and 10 cm to the distal side within a colon resection, such as that partially shown in Figure 13 (a ruler is present for measurements). For resections with large TVAs, we took samples 1 cm and 10 cm from the tumors. Thus, about 18 cm in length of colonic mucosa for cancer resections and about 9 cm in length for TVAs were sampled.

**Table 1:** Percent of crypts which showed high expression of Pms2, Ercc1 or Ku86

Patient designation C = cancer A = adenoma	Total crypts evaluated (# of tissue samples)	% Pms2 high in each tissue section	% Ercc1 high in each tissue section	% Ku86 high in each tissue section
C1	958 (6)	22,15,42,1,22,13	40,18,19,29,10,16	98,100,93,96,99,100

C2	921 (5)	32,31,9,1,17,-	26,13,25,10,13,-	99,98,100,99,99,-

C3	968 (6)	33,87,5,1,1,0	91,5,92,21,11,97	N.D.

C4	2,411 (6)	1,4,0,0,1,8	5,11,9,8,11,18	N.D.

C5	448 (4)	-,0,0,0,-,0	-,0,4,12,-,15	N.D.

C6	1,801 (5)	7,1,32,-,11,0	38,87,18,-,1,6	N.D.

C7	2,844 (6)	2,58,43,2,2,3	-,52,45,48,70,52	N.D.

C8	1,742 (6)	1,3,1,0,1,2	4,5,10,6,13,8	97,97,97,100,99,99

A1	433 (2)	14,19	7,3	99,-

A2	195 (2)	2,16	16,20	79,75

A3	90 (2)	0,0	2,27	100,-

A4	112 (2)	0,0	24,14	92,87

A5	129 (2)	0,0	10,24	64,98

A6	72 (2)	0,3	15,18	89,89

A7	255 (2)	5,9	37,29	94,48

A8	203 (2)	0,0	15,29	100,95

Percent of crypts with high expression in cancer resections are shown in order, from left to right, for 10, 3 or 1 cm proximal to and for 1, 3 or 10 cm distal to the cancer. Percent of crypts with high expression in tubulovillus adenoma resections are shown in order 10 cm and 1 cm from the tumor. A dash indicates that the tissue sample was not available for immunostaining. N.D. indicates Not Done

Tissue sections were cut from these tissue samples, immunostained for Pms2, Ercc1 and Ku86, and evaluated for percent of crypts that had high expression. The number of crypts evaluated for each patient is shown in Table 1. A streaming video, as a microscope is used to pan through a tissue section while crypts are evaluated for level of expression, is shown in the public access video publication by Nguyen et al. [35] at minutes:seconds 17:40 to 21:51. In this segment of the video, 33% of crypts had high Ercc1 expression and 67% of crypts had reduced expression. The next section of the video shows a tissue section with only 1% of crypts (a single crypt) with high expression of both Ercc1 and Pms2 at minutes:seconds 21:51 to 23:50. A following segment of this video shows evaluation of Ku86 in a tissue section. (Viewing of different segments of this video is best done after downloading the entire 345 MB video.)

**Figure 14:**
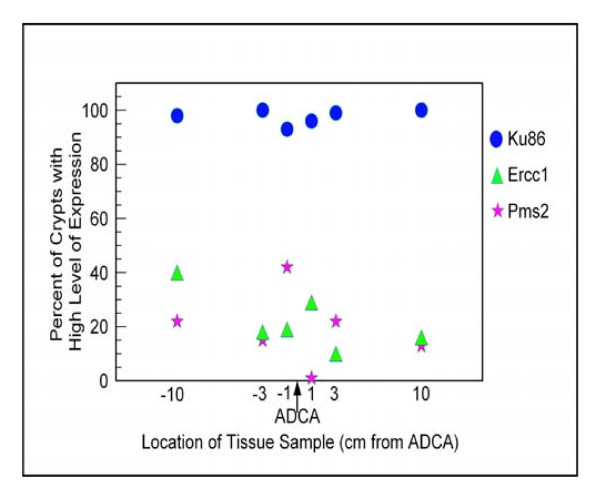
**Percent of crypts with high expression of Pms2, Ercc1 or Xpf**. Tissue samples were taken at the indicated distances from a colon cancer. The distances are -10, -3 or -1 cm on the proximal side and 1, 3 and 10 cm on the distal side of a resection from patient C1 (from Table 1). The symbols indicate the percent of crypts showing high expression of Pms2, Ercc1 or Ku86 in each tissue sample.

The frequency of crypts with high expression for each protein is shown in Table 1. From 16 patients, 4 of 60 samples, or 7%, had high expression of either Pms2 or Ercc1 (more than 85% of crypts with high expression). In contrast, 28 of 32 samples, or 88%, had high expression of Ku86. A graphical representation of the results from patient C1 is shown in Figure 14.

**Figure 15:**
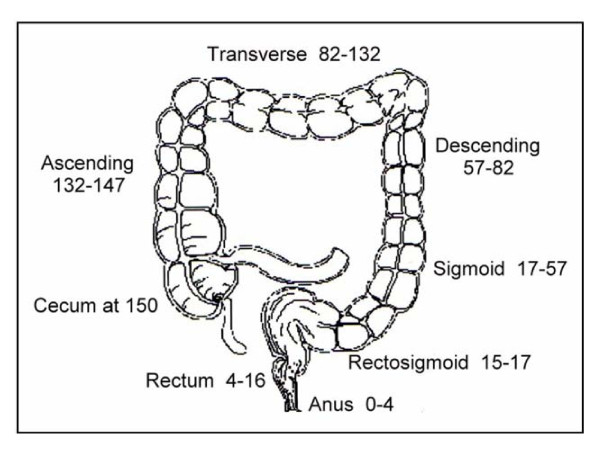
**Diagram of the human lower gastrointestinal tract**. This diagram illustrates the locations of the regions referred to in Table 2. Distances from the anal verge are shown in centimeters.

A diagram of the human lower gastrointestinal tract is shown in Figure 15. The colon is considered to be the areas of ascending, transverse and descending/sigmoid colon. In the standard diagram shown in Figure 15, the total length of the lower gastrointestinal tract is represented as 150 cm, with the colon (from the cecum to the rectosigmoid junction) being 130 cm. This is consistent with the lengths of colon measured in living individuals (reviewed in [36]). From our observations of 32 resected colon segments, with 12 in the ascending colon, 6 in the transverse colon and 14 in the descending/sigmoid colon (Table 2) and taking an average of the inner circumferences of the three regions, we determined that 6.2 cm is the average inner circumference of the colon (see Figure 13 for how inner circumferences were measured, after colon segments were opened longitudinally).

**Table 2:** Inner circumference* of regions of the lower gastrointestinal tract

Region of lower GI tract	Number of resections measured	**Mean inner circumference +/- S.E.M**.	Range of values
Cecum	12	8.7 +/- 0.3 cm	8-10.5 cm

Ascending colon	12	6.6 +/- 0.1 cm	6-7 cm

Transverse colon	6	5.8 +/- 0.3 cm	5-6.5 cm

Descending/Sigmoid colon	14	6.3 +/- 0.1 cm	6-6.8 cm

Rectum near rectal/sigmoid junction	12	5.7+/- 0.4 cm	4.5-7.5 cm

*Measured within 20-30 minutes of removal from the body

From the images of colonic mucosa in reference [12], where tissue sections are cut either through the short axes of crypts or through their long axes, and the images include reference size bars, we see that there are about 100 crypts per sq mm in the colonic epithelium. The inner longitudinal dimension of the freshly obtained, non-fixed human colon (from 100 male and 100 female cadavers, measured a few hours after being brought to the laboratory, or after the intestinal tracts were preserved in a refrigerator at 3°C for 24-72 hours) is, on average, 160.5 cm (measured from the bottom of the cecum to the colorectal junction) with a range of 80 cm to 313 cm [36]. The intestine lengthens after death, possibly because of loss of intestinal muscular tone (reviewed in [36]). Thus, a 160.5 cm length is consistent with the length shown in Figure 15, representing the living colon. Using the length of 160.5 cm and an average of 6.2 cm for the inner circumference of the colon, the inner mucosa would have a surface area, on average, of about 995 sq cm, or about 9,950,000 (close to 10 million) crypts. The crypts are connected through further epithelial cells at their open ends, forming a microscopically indented epithelial sheet (the indents being the crypts), covering the average 160.5 by 6.2 cm inner surface area.

We sampled tissues from -10 to -1 and from 1 to 10 cm on the proximal and distal sides, respectively, of colon cancers of 8 patients and tissues from 1 cm to 10 cm distant from TVAs. In cancer resections, we sampled about 18 cm of length (about 11%) of the total lengths of the colons. This would be an area having a little more than 1 million crypts. Thus, it appears that a field defect comprising about 1 million crypts, with most crypts expressing reduced protein levels of Pms2 and Ercc1, but high levels of Ku86, surrounds the cancers of these patients, and likely similar field defects surround TVAs as well.

## Discussion

### Pms2 deficiency is not generally due to mutation or protein degradation

It appears unlikely that most cases of reduced expression of Pms2 protein that we observed are due to somatic mutation of the Pms2 gene. Somatic mutations in DNA repair genes are infrequent in sporadic cancers, and no Pms2 or Xpf mutations were found in Pms2 and Xpf gene sequences of 11 colon cancers [37]. Similarly, in 119 cases of tumors classified as mismatch repair deficient and lacking Pms2 expression reported by Truninger et al. [38], Pms2 was deficient in 103 because of lack of its pairing partner Mlh1. Pairing of Pms2 with Mlh1 gives Pms2 its stability [23]. The Mlh1 loss, for sporadic cancers, was due to epigenetic silencing caused by promoter methylation in 65 out of 66 cases. In 16 cancers Pms2 was deficient when Mlh1 protein expression was present. Of these 16 cases, no cause was determined for 10, but 6 were found to have a heterozygous germline mutation in Pms2, followed by likely loss of heterozygosity in the tumor. Thus only 6 of 119 tumors lacking expression for Pms2 (5%) were due to mutation in Pms2.

As pointed out in the Background section, it has been suggested that ROS-caused reduced expression of Pms2 and Ercc1 was due to protein degradation. If this were so, then Pms2 and Ercc1 would be reduced in a coordinated manner. However, when tissue samples were deficient in Ercc1, only 66% of those samples were also deficient in Pms2. Figure 7, where expression of Pms2 was reduced and Ercc1 was expressed at a high level, illustrates discordance. Thus it seems unlikely that Pms2 and Ercc1 are reduced primarily due to ROS-caused protein degradation.

### Most instances of Pms2 deficiency in colon cancers are due to an epigenetic alteration

As indicated in the previous section, mismatch repair protein Mlh1 interacts with Pms2. They form the heterodimer MutLα that is essential for mismatch repair. In the absence of its Mlh1 partner, Pms2 is unstable and is degraded [23]. As also noted above, when Pms2 was reduced in 119 tumors, for 103 (87%) of the tumors the human *mlh1 *gene was transcriptionally silenced by promoter methylation [38]. The microRNA, miR-155, is over-expressed in colon tumors as well, and also down-regulates Mlh1 [39], which in turn would cause reduced expression of Pms2. As reviewed by Valeri et al. [39], miRNA transcription levels are frequently controlled by epigenetic factors including DNA methylation (by DNA methyltransfereases or DNMTs), histone modification (by histone methylation or histone deacetylases or HDACs), or by the polycomb repressive complex 2 (PRC2). Thus, the majority of instances of reduced Pms2 are likely due to epigenetic alterations.

### Ercc1 and Xpf deficiencies are not likely due to mutation or protein degradation

In the present study, deficient expression of Ercc1 follows a pattern where the cells near the top of individual crypts recover from the deficiency. Xpf has a similar, though less pronounced pattern of expression. These observations are inconsistent with a somatic mutation in the crypt cell lineage, where the stem cells are at the base of the crypts and their descendents progressively move towards the top of the crypt [5,6]. In addition, as noted above, no Pms2 or Xpf mutations were found in Pms2 and Xpf gene sequences of 11 colon cancers [37].

It also seems unlikely that the reduced expression of Ercc1 and Xpf that we observed would be due to post-translational modifications of the proteins, such as degradation of the proteins by ROS. While Pms2 and Ercc1 have both been shown to be subject to degradation by ROS, 34% of the tissue samples deficient for Ercc1 were not deficient for Pms2. This lack of consistent simultaneous deficiency in Pms2 and Ercc1 indicates that, in the tissues we evaluated, protein degradation due to ROS is not likely to be the primary source of reduced expression.

### Ercc1, and possibly Xpf, deficiency is likely due to an epigenetic alteration

Reduction in Ercc1 protein expression could be caused by an epigenetic mechanism. A TAR miRNA, coded for by the HIV virus, down-regulates Ercc1 protein expression [40]. TAR miRNA allows Ercc1 mRNA to be transcribed, but acts at the p-body level to prevent translation of Ercc1 protein. (A p-body is a cytoplasmic granule "processing body" that interacts with miRNAs to repress translation or trigger degradation of target RNAs.) A survey of miRNA homology regions to human Ercc1 mRNA [41] indicated at least 21 miRNAs had regions of homology to Ercc1 mRNA that could act to decrease Ercc1 mRNA translation (Xpf had no reported homologous miRNA). As noted above, a review by Valeri et al. [39] indicated that miRNA transcription levels are frequently controlled by epigenetic factors.

We observed that cancers frequently had areas with restored expression of Pms2, Ercc1 and/or Xpf, even though surrounding areas of the cancers had reduced levels of Pms2, Ercc1 and/or Xpf. This could indicate selection of sub-clones for which epigenetically reduced expression of Pms2, Ercc1 and/or Xpf was reversed under the new micro-environment of a growing cancer.

### Elimination of factors at the transcriptional level, or protein turnover level, that might reduce protein expression of Ercc1

McGurk et al. [42] examined and eliminated a number of factors that might control protein expression levels of Ercc1. They first established that Ercc1 mRNA could occur as either the wild-type sequence or in three splice variants (one generating a larger protein than wild-type). Each of the splice variants could produce proteins lacking the Xpf binding region. Ercc1 mRNA was also found to have either wild-type or three alternative transcription start points. Neither the level of overall mRNA transcription, splice variation nor transcription start point of mRNA correlated with protein level of Ercc1. Rate of Ercc1 protein turnover also did not correlate with Ercc1 protein level. Ercc1 transcripts with longer or shorter 5'-UTRs were, as well, not responsible for levels of Ercc1 protein. They concluded that control of Ercc1 protein level occurred at the translational level.

### Coordination, and non-coordination, of protein levels of Ercc1 and Xpf

Explanations are needed both for when Ercc1 and Xpf have coordinated lack of expression in nuclei, and also for when their protein expression is discordant (Figure 7 and Figure 12).

Early reports indicated that Ercc1 was not stable in the absence of Xpf [43,44], and similarly, Xpf was reported to be unstable in the absence of Ercc1 [44]. More recent results modified these observations, since knockdown of Xpf mRNA expression by siRNA (and consequent loss of Xpf protein expression) did not eliminate protein expression of Ercc1 in a human cancer cell line [45]. However, the location of the Ercc1 expression in the cells, under conditions of the siRNA knockdown of Xpf was not determined, so it is not clear whether the continued expression of Ercc1 resulted in nuclear location of the Ercc1. In our open access video publication [35] at minutes:seconds 11:35 to 12:43, we illustrate that when Ercc1 is low in the nucleus it may build up in the cytoplasm. Ercc1 was first thought to have its own nuclear localization signal (NLS) (note added in proof to [46]). Since Ercc1 had one NLS but did not have a cluster of NLSs, it was later suggested that Ercc1 may depend upon its binding with Xpf for its nuclear import, since Xpf appeared to have 4 NLSs [47]. A recent report by Ahmad et al. [48] found that mutant Xpf protein could bind to Ercc1 and prevent Ercc1 (and Xpf) from entering the nucleus. From these reports, the simultaneous reduced or absent protein expression of Ercc1 and Xpf in the cell nucleus could be expected. Consistent with these reports, we found that for 81 tissue samples deficient in Ercc1, 43 (53%) were also deficient in Xpf expression. In the reverse evaluation, when 53 samples were deficient for Xpf, 43 (81%) of these samples were also deficient for Ercc1.

However, our results showed that 31 out of 81 (47%) of tissue samples deficient in Ercc1 still had high nuclear expression of Xpf, and 10 out of 53 (19%) tissue samples deficient in nuclear Xpf still had high nuclear expression of Ercc1.

One possible reason for why protein expression of Ercc1 and Xpf can be discordant may be that the cells in which Ercc1 and Xpf were shown to be unstable in the absence of each other were not of colonic epithelial origin [43,44]. It may be that Ercc1 and Xpf have other pairing partners that are sometimes high in cells of colonic epithelial origin, and in such cells could give Ercc1 or Xpf increased stability, as well as provide a NLS for bringing Ercc1 or Xpf into the nucleus.

As pointed out by Dingwall and Laskey [49], a protein without a NLS can still be transported into the nucleus as part of a complex with a protein that has a nuclear targeting sequence. Thus, Xpf interacts strongly with Pcna [50,51] and Pcna has a nuclear locator signal (NLS) [52]. The Fancg protein has strong affinity for Ercc1 and moderate affinity for Xpf [53]. Fancg, when also complexed with Fanca, is transported to the nucleus [54]. Ercc1 and Xpf each bind to Msh2 [55]. Msh2 has two NLSs [56]. Ercc1 has a binding domain for Xpa [57,58], and Xpa has a NLS [59]. Therefore, it is possible that, in some tissues progressing to cancer, one or more of these proteins that interact with Ercc1 and/or Xpf may provide both stability and nuclear transport for one member of the pair when the other is reduced or absent.

### Coordination, and non-coordination, of protein levels of Pms2 with Ercc1 and Xpf

Deficiencies in expression of Pms2 are either coordinated or un-coordinated with deficiencies in Ercc1 and/or Xpf. This is indicated in Figures 4, 5, 6, 7, 11 and 12. For 63 samples deficient for Pms2, 56 (89%) were also deficient for Ercc1 and for 85 samples deficient for Ercc1, 56 (66%) were also deficient in Pms2 expression. Deficiency in Ercc1 and Xpf likely correlates with deficiency in Pms2 because a defect in Pms2 (causing apoptosis resistance) would be selected for in the face of the increased DNA damages accumulated when Ercc1 and/or Xpf are deficient. This is supported by the results of Nara et al. [34], who showed that when Ercc1 deficient Chinese hamster ovary cells were repeatedly subjected to DNA damage, of five clones derived from the surviving cells, three were mutated in Pms2.

The fact that a deficiency of Ercc1 and/or Xpf is sometimes present without a Pms2 deficiency may also be explained by the results of Nara et al. [34]. The other two clones derived from surviving cells, in the experiment above, were mutated in Msh2 and Msh6, two other mismatch repair proteins that also have essential roles in apoptosis (reviewed in [20]). Deficiencies in one of these proteins could be selected for, rather than Pms2, to protect cells deficient in Ercc1 or Xpf (and thus accumulating DNA damages) from apoptosis.

### Likely role of deficiencies in Pms2, Ercc1 and Xpf in progression to colon cancer

In the study of Nara et al. [34], *ercc1, pms2 *double mutant Chinese hamster ovary cells, when exposed to UV light (a DNA damaging agent), showed a 7,375-fold greater mutation frequency than wild-type Chinese hamster ovary cells, and a 967-fold greater mutation frequency than the cells defective in Ercc1, alone. Thus colonic cells deficient in both Ercc1 and Pms2 would be genetically unstable. A similar genetically unstable situation would be expected for cells doubly defective for Pms2 and Xpf. This instability would be expected to enhance progression to colon cancer by causing a mutator phenotype [60], and could also account for the presence of the cells doubly deficient in Pms2 and Ercc1 (or Xpf) we observed in field defects associated with colon cancer.

### Alterations in other apoptosis proteins appear to be later events in progression to colon cancer

Both deficiency of CcOI and increased Maspin protein expression appear to cause apoptosis resistance, as reviewed for CcOI by Bernstein et al. [12], and shown for Maspin by Payne et al. [61]. Immunohistochemical evaluations were made for deficiency of cytochrome coxidase subunit I (CcOI) [12] or increased expression of Maspin [61] in colonic epithelium. These evaluations were made in tissues from biopsies of patients with no history of colonic neoplasia, from tissue samples taken from resections 1-10 cm distant from colon cancers, tissues at the margins of cancers and tissue samples within cancers (and for Maspin, also within adenomas). As noted above, resistance to apoptosis may allow cells with DNA damage to survive, leading to increased chromosomal instability and, potentially, progression to colon cancer.

CcOI is coded for by mitochondrial DNA and CcOI protein deficiency of a crypt is known to arise from mutation of the *CcOI *gene [10]. While there was increased frequency of crypts with CcOI deficiency in tissues 1-10 cm from colon cancers, there were no large patches of crypts with CcOI deficiency in these tissues. Only in tissues at the margins of crypts were large CcOI-deficient patches, with several hundred adjacent CcOI-deficient crypts, observed [12]. That is, in Figure 1, large CcOI-deficient patches would occur in the irregular circle just outside of the irregular circle denoting a cancer. This indicates that colon cancers may arise from CcOI deficient patches of crypts, but this CcOI deficiency may occur and grow into a large patch at a later stage in progression to colon cancer than deficiency for Pms2, Ercc1 or Xpf, which may occur in as many as 1 million crypts surrounding colon cancers.

Strongly increased maspin protein expression was only seen in polyps or in colon cancers, but not in tissues surrounding cancers [61]. Thus, increased maspin expression is likely among the last changes in formation of a colon cancer.

## Conclusions and implications

Overall, substantial deficiencies in protein expression of DNA repair proteins Pms2, Ercc1 and Xpf frequently occur in a coordinated manner in extensive regions, involving as many as 1 million crypts, near cancers, and also occur within cancers. This suggests that colon cancers tend to arise in field defects that are deficient in DNA repair and that deficiencies in protein expression of Pms2, Ercc1 and Xpf are frequent early, and often coordinated, steps in progression to colon cancer, as well as further progression within cancers.

Our results suggest that blocking any one oncogenic pathway in a colon cancer may only slow down further progression, if the cancer was already deficient in DNA repair. Reduced DNA repair capability combined with increased apoptosis resistance in a cancer, and any metastasized cells from the cancer, would tend to increase DNA damages that give rise to further mutations after DNA replication, likely unleashing other pathways of progression.

Our findings also suggest that the current therapeutic approach to treating colon cancer, with surgical removal of the cancer and a good part of the surrounding field defect, is fairly effective, since it would remove many of the secondary and tertiary mutations leading to the cancer. However, this could be followed by use of therapeutic DNA damaging agents. Remaining parts of the field defect (and any metastasized cells) would be more deficient in DNA repair than the surrounding normal areas of the colon, outside the field defect.

In addition, it was recently found that inhibition of an additional DNA repair enzyme - such as inhibition of the repair enzyme PARP in breast cancer - facilitates killing of tumor cells [62]. Similar further DNA repair inhibition in the case of colon cancer could also be pursued. This may cause repair deficient cells in a remaining portion of a field defect, or in metastasized cells, to be even more susceptible to the killing effects of therapeutic DNA damaging agents.

## Methods

### Tissue procurement

Before any biopsy tissue samples were obtained during colonoscopy, informed consent was given by the patient, using a form approved by the University of Arizona Institutional Review Board. Biopsied colonic mucosal samples were fixed in 10% buffered formalin for 4 hours, then transferred to 70% alcohol, followed by paraffin embedment. Tissue samples from colonic resections were obtained after informed consent before surgery, and these larger tissue samples were fixed in 10% buffered formalin for 24 to 36 hours, then transferred to 70% alcohol, followed by paraffin embedment.

Overall, tissue biopsies were taken during colonoscopies of 77 patients at 4 different risk levels for colon cancer, including 19 patients who had never had colonic neoplasia (who served as controls). In addition, 158 tissue samples were taken from tissues near or within colon cancers removed by resection and 16 tissue samples were taken near tubulovillous adenomas (TVAs) removed by resection. A total of 568 triplicate tissue sections (a total of 1,704 tissue sections, obtained as described below) from these tissue samples were evaluated by immunohistochemistry for one of 4 DNA repair proteins.

### Immunohistochemical procedure

The general procedures used were demonstrated in the first 8 minutes of our methods video publication [35]. Briefly, 4 μm sections were cut from formalin-fixed and paraffin-embedded tissues and placed on slides. Tissue sections for staining with Pms2, Ercc1 and Xpf were placed on slides so that cuts 1, 4 and 7 were placed on the slide for Pms2, cuts 2, 5 and 8 were placed on the slide for Ercc1, and cuts 3, 6 and 9 were placed on the slide for Xpf. For staining with Ku86, three sequential cuts were placed on one slide. These multiple tissue sections were used in order to be sure that areas with absent staining were not due to bubbles that may have prevented the antibodies from reaching a given tissue area, and to allow comparisons of at least two tissue sections even when one of the three tissue sections on a slide may have had a fold in an area. Sections were deparaffinized with Xylene and graded ethanols, then rehydrated.

Antigen retrieval was performed by microwave exposure in VECTOR Antigen Unmasking Solution (obtained from Vector Laboratories, inc., Burlingame, CA) according to the manufacturer's instructions. The slides were then rinsed with distilled water.

Endogenous peroxidase activity was blocked by incubation in 3% hydrogen peroxide in methanol for 20 min, and then the tissue sections were rinsed with distilled water, "TBST" buffer [1 ml Tween + 100 ml 10x Tris + 900 ml distilled water (where 10x Tris is 24.2 grams Trizma + 80 grams NaCl + 1,000 ml distilled and deionized water + concentrated HCl {approximately 15 ml} to bring the solution to pH 7.6)], and PBS. The TBST step is not used with the Ercc1 or Ku-86 protocol.

Slides were placed in Sequenza staining racks (Shandon Sequenza Immunostaining System from Thermo Scientific, Thermo Fisher Scientific Inc., Waltham, MA) and rinsed with PBS. For ERCC1, PMS2 and XPF, 3 drops/slide of "Background Sniper" (from Biocare Mach 3 kit, Biocare Medical, Concord CA) were added and left for 10 min at room temperature (RT) to reduce non-specific staining of background proteins. For Ku86, 150 ul of 1.5% normal goat serum in 2% BSA/PBS was used for one hour. The slides were rinsed with TBST (PBS was used instead of TBST for Ercc1).

A primary mouse monoclonal antibody was used for Pms2 (Pharmingen, Becton, Dickinson and Company, San Diego CA); Ercc1 (8F1 from Neomarkers, Freemont CA); or Xpf (3F2/3 obtained from Abcam plc. San Francisco, CA). Ku-86 (H-300 obtained from Santa Cruz Biotechnology, inc., Santa Cruz, CA) is a rabbit polyclonal antibody.

125 ul primary antibody was added as follows: Pms2 mouse monoclonal antibody was added at 10 μg/ml in 2% BSA/TBST mixture and left to incubate at RT for 2 hours before 3 TBST rinses were applied. Ercc1 mouse monoclonal antibody was added at 2 μg/ml in 2% BSA/PBS and left to incubate at room temperature for 45 minutes before 3 PBS washes. Xpf mouse monoclonal antibody was added at 5 μg/ml in Renoir Red (from Biocare Mach 3 kit, Biocare Medical, Concord CA), and incubated for 1 hour at room temperature, followed by 3 TBST rinses. Ku-86 primary rabbit polyclonal antibody was added at 2 ug/ml diluted in 2% normal goat serum in 2% BSA/PBS and incubated for 1 hour at room temperature, followed by 3 TBST rinses.

Secondary antibody was added as follows: For Pms2, the polyclonal rabbit anti-mouse Dako Biotinylated secondary antibody (E0413, DAKO Corp., Carpinteria, CA) was added at 120 μl/slide at a 1:100 dilution (in 2% BSA/TBST) to the slides and incubated for 30 minutes at room temperature before being rinsed 3 times with TBST.

For Ercc1, the same Dako secondary antibody as above was added to the slides at 120 μl/slide at a 1:300 dilution (in 2% BSA/PBS) and incubated for 30 minutes before 3 PBS washes. The Dako secondary antibody was not used for Xpf. For Xpf, Mouse Probe and Mouse Polymer (Biocare Mach 3 kit, Biocare Medical, Concord CA) were used instead of Dako secondary antibody. Mouse Polymer HRP was added at 4 drops/slide for 15 min, followed by TBST and PBS rinses. For Ku-86, Goat anti-rabbit biotinylated IgG secondary antibody was used (BA-1000 Vector Laboratories, inc., Burlingame CA) at a 1:100 ratio diluted into 2% BSA/PBS at 120 ul/slide for 30 minutes, followed by 3 PBS rinses.

Vectastain Elite avidin-biotin complex method kit PK 6100 (Vector Laboratories, inc., Burlingame, CA) was then used according to the manufacturer's instructions in the Pms2, Ercc1, and Ku-86 protocols at 3 drops/slide and incubated at room temperature for 30 minutes before 2 rinses with TBST(for Pms2) and PBS (for Ercc1 and Ku86). For XPF, four drops/slide of Mouse Probe were added for 15 min, followed by 3 TBST rinses.

The slides were then removed from the Sequenzas, and color development was carried out by applying .025% diaminobenzidine tetrachloride (Sigma, St. Louis, MO) in PBS supplemented with 0.04% hydrogen peroxide. Sections were counterstained with 1:4 diluted hematoxylin (Sigma), dehydrated in a graded series of ethanols followed by xylene, and then mounted with coverslips using Cytoseal XYL (Richard Allen Scientific, Kalamazoo, MI). Brown staining indicates Pms2, Ercc1, Xpf, or Ku-86 expression, and blue staining from hematoxylin identifies nucleoproteins in the nucleus. Table 3 briefly outlines the steps used for IHC of the four proteins in a tabular form, to make the four different protocols more easily followed.

**Table 3:** Tabular form of IHC

Pms2	Ercc1	Xpf	Ku86
Cut 4 micron sections	Cut 4 micron sections	Cut 4 micron sections	Cut 4 micron sections

Deparaffinize and rehydrate	Deparaffinize and rehydrate	Deparaffinize and rehydrate	Deparaffinize and rehydrate

Antigen retrieval in microwave with VECTOR Antigen Unmasking Solution	Antigen retrieval in microwave with VECTOR Antigen Unmasking Solution	Antigen retrieval in microwave with VECTOR Antigen Unmasking Solution	Antigen retrieval in microwave with VECTOR Antigen Unmasking Solution

Rinse with PBS and distilled water	Rinse with PBS and distilled H_2_O	Rinse with PBS and distilled water	Rinse with PBS and distilled water

Incubate in 3% H_2_O_2 _in methanol for 20 min	Incubate in 3% H_2_O_2 _in methanol for 20 min	Incubate in 3% H_2_O_2 _in methanol for 20 min	Incubate in 3% H_2_O_2 _in methanol for 20 min

Rinse in distilled water, PBS	Rinse in distilled water, PBS	Rinse in distilled water, TBST, PBS	Rinse in distilled water, PBS

Place in Sequenza racks, rinse with PBS	Place in Sequenza racks, rinse with PBS	Place in Sequenza racks, rinse with PBS	Place in Sequenza racks, rinse with PBS

Add 3 drops Background Sniper, 10 min at room temp	Add 3 drops Background Sniper, 10 min at room temp	Add 3 drops Background Sniper, 10 min at room temp	Add 1.5% normal goat serum in 2% BSA/PBS, 120 μl, one hour

Rinse with TBST	Rinse with PBS	Rinse with TBST	Do Not Rinse

Primary mouse monoclonal from Pharmingen at 10 μg/ml into 2% BSA/TBST, 120 μl/slide	Primary mouse monoclonal 8F1 from Neomarkers at 2 μg/ml with 2% BSA/PBS, 120 μl/slide	Primary rabbit polyclonal 3F2/3 from Abcam at 5 μg/ml in Renoir Red at 120 μl/slide	Primary mouse monoclonal H-300 from Santa Cruz at 2 μg/ml in 2% BSA/PBS, 120 μl/slide

Incubate 2 hrs at room temp	Incubate 45 min at room temp	Incubate 1 hour at room temp	Incubate 45 min at room temp

3 rinses with TBST	3 rinses with PBS	3 rinses with TBST	3 rinses with PBS

Polyclonal rabbit anit-mouse antibody Dako Biotinylated secondary antibody at 100 μl/slide at 1:100 dilution in 2% BSA in TBST	Polyclonal rabbit anit-mouse antibody Dako Biotinylated secondary antibody at 100 μl/slide at 1:300 dilution in 2% BSA/PBS	Mouse Probe (Biocare) 4 drops/slide	Goat anti-rabbit biotinylated IgG secondary antibody at 1:100 dilution in 2% BSA/PBS, 100 μl/slide

Incubate 30 min at room temp	Incubate 30 min at room temp	Incubate 15 min room temp	Incubate 30 min at room temp

3 rinses with TBST	3 rinses with PBS	3 rinses with TBST	3 rinses with PBS

Vectastain Elite avidin-biotin complex method kit 3 drops/slide	Vectastain Elite avidin-biotin complex method kit 3 drops/slide	Mouse Polymer (Biocare) 4 drops/slide	Vectastain Elite avidin-biotin complex method kit 3 drops/slide

Incubate 30 min room temp	Incubate 30 min room temp	Incubate 15 min room temp	Incubate 30 min room temp

2 rinses TBST, then PBS	2 rinses PBS	3 rinses TBST, then PBS	2 rinses PBS

Diaminobenzidine tetrachloride plus 0.04% H_2_O_2_	Diaminobenzidine tetrachloride plus 0.04% H_2_O_2_	Diaminobenzidine tetrachloride plus 0.04% H_2_O_2_	Diaminobenzidine tetrachloride plus 0.04% H_2_O_2_

Counterstain with hematoxylin	Counterstain with hematoxylin	Counterstain with hematoxylin	Counterstain with hematoxylin

Dehydrate with ethanols followed by xylene	Dehydrate with ethanols followed by xylene	Dehydrate with ethanols followed by xylene	Dehydrate with ethanols followed by xylene

Mount with Cytoseal and coverslips	Mount w/Cytoseal and coverslips	Mount with Cytoseal and coverslips	Mount with Cytoseal and coverslips

### Quantitation of high or reduced expression of Pms2, Ercc1, Xpf, and Ku86

After being prepared using the immunohistochemical procedure, slides were viewed with a Motic BA300 digital photomicroscope, and the level of immunohistochemical staining was evaluated both directly with the microscope and with a digital image on a computer monitor, using Motic Images Plus version 2.0 software. The software was set with Gain 0, Offset 0, Enhance disabled, Gamma disabled, R,G,B of gain 1, brightness 0, Edge Detection disabled, Sharpness disabled, Resolution 1024/768, White Balance on, Auto Exposure on and then switched off with an increase in brightness sufficient to allow background (in areas of the slide without tissue present) to become white or nearly white.

The colonic crypts were divided into colonic lumen surface, crypt neck, and crypt body, as shown in the Results (Figure 3). Colonic crypts are composed primarily of absorptive cells and goblet cells. Goblet cell nuclei had variable levels of expression of the proteins of interest, while absorptive cell nuclei of the bodies of crypts were consistent in expression of the proteins of interest from the stem cell region at the base of the crypts to the neck region of crypts. For the semi-quantitative values shown in Figures 10, 11 and 12 for Pms2, Ercc1 or Xpf, frequencies of high expression of the proteins of interest in absorptive cell nuclei of the bodies of crypts were assessed for the entire piece of tissue seen in the tissue section. Thus, a value of 20% assigned to a tissue section would indicate that 20% of the absorptive cell nuclei in the bodies of crypts had high expression for the protein being evaluated (there were usually 30-150 identifiable body sections of crypts seen per tissue section). For values of Ku86 shown in Figures 10, 11 and 12, the values are expressed as percent of entire crypts showing high expression of the protein, since Ku86 protein expression was either high or very low in individual crypts.

For the values of Ercc1 and Pms2, shown in Table 1 and Figure 14, a different method was used, where the percent of crypts having high expression of the protein was evaluated. Crypts were labeled 0, 1, 2, 3 or 4 for absent expression, just detectable expression, low expression, medium high expression or highest expression, respectively. Those crypts labeled 3 or 4 were considered to have high expression, and those labeled 0, 1 or 2 were considered to have deficient expression. This method, for a tissue section with 33% of crypts having high expression for Ercc1, is illustrated in the public access video publication by Nguyen et al. [35] at minutes:seconds 17:40 to 21:51. The next section of this video shows a tissue section with very low expression of both Ercc1 and Pms2 in about 100 crypts but with one crypt with high expression of both proteins, at minutes:seconds 21:51 to 23:50 of the 28 minute video. The 345 MB Mpeg4 video can be best downloaded from the PubMed Central web site, in order to access specific minutes, and have the ability to replay those sections, without viewing the entire video.

### Statistical analysis

Boxplots, scatter plots and means and standard errors of the means (Figures 10 and 11 and Table 2, respectively) were made using SPSS 17.0 software.

### Photomicrographs

All micrographs were obtained with a Motic BA300 digital photomicroscope with integrated camera, using Motic Images Plus version 2.0 software adjusted as described above. The images were then saved as tif images, and adjusted in Paint Shop Pro 5 to maximize brightness and contrast between brown staining representing expression of the protein being evaluated and the blue color representing hemotoxylin staining of DNA.

## Competing interests

The authors declare that they have no competing interests.

## Authors' contributions

CB designed the experiments, RSK and BB obtained the tissues and provided pathology information on patient progression to cancer, ARP provided histological and pathology analysis of tumors and adjacent tissues, LR devised reproducible and robust immunohistochemical methods, AF, HN, CL devised standard methods for semi-quantitative immunohistochemical evaluation of protein expression, AF, HN, CL, SK, SS, NO performed extensive evaluations of expression of the proteins and took the illustrative digital photo images, BZ carried out the statistical analyses, CB produced the diagrams, ARP and VN provided measurements of internal circumferences of colon segments, CB wrote the manuscript, HB, BZ and CMP provided critical editing of the manuscript. All the authors approved the final version of the manuscript.
